# Expression of K6W-ubiquitin in lens epithelial cells leads to upregulation of a broad spectrum of molecular chaperones

**Published:** 2008-03-04

**Authors:** Q Bian, AF Fernandes, A Taylor, M Wu, P Pereira, F Shang

**Affiliations:** 1 USDA HNRCA at Tufts University, Boston, Massachusetts; 2 Zhongshan Ophthalmic Center, Sun Yat-sen University, Guangzhou, China; 3 Center of Ophthalmology, IBILI – Faculty of Medicine, University of Coimbra, Coimbra, Portugal

## Abstract

**Purpose:**

Accumulation and precipitation of abnormal proteins are associated with many age-related diseases. The ubiquitin-proteasome pathway (UPP) is one of the protein quality control mechanisms that selectively degrade damaged or obsolete proteins. The other arm of the protein quality control mechanism is molecular chaperones, which bind to and help refold unfolded or misfolded proteins. We previously showed that the molecular chaperones and the UPP work in a competitive manner in eliminating the denatured proteins. To further investigate the interaction between the two protein quality control mechanisms, we determined the effects of the impairment of the UPP on the expression of molecular chaperones in human lens epithelial cells (HLEC).

**Methods:**

K6W-ubiquitin, a dominant negative inhibitor of the UPP, was expressed in confluent HLEC via an adenoviral vector. The mRNA levels of cytoplasmic and endoplasmic reticulum (ER) chaperones were determined by real-time reverse transcription polymerase chain reaction (RT–PCR). Protein levels for these chaperones were determined by western blotting.

**Results:**

Expression of K6W-ubiquitin in HLEC increased the expression of a broad spectrum of molecular chaperones. Among the heat-shock proteins, mRNA for αB-crystallin, Hsp70, and Hsp90 increased 27 fold, 21 fold, and twofold, respectively, in response to K6W-ubiquitin expression. Among the ER chaperones and ER stress related factors, mRNA levels of protein disulfide isomerase, Grp75, Grp78, Grp94, and the CAAT/enhancer binding protein homologous protein (CHOP) increased from 1.7 fold to 3.7 fold. The mRNA for Hsp60 also increased 1.6 fold in response to the expression of K6W-ubiquitin. The expression pattern of these chaperones in response to the expression of K6W ubiquitin is similar to that obtained when cells were treated with proteasome inhibitors or heat-shock.

**Conclusions:**

It appears that the upregulation of these chaperones is related to the elevated levels of abnormal proteins in the cells. These findings support our hypothesis that the molecular chaperones and the UPP may back each other up in the process of protein quality control. The upregulation of molecular chaperones in response to the expression of a dominant negative ubiquitin may compensate for the impairment of the UPP in the degradation of abnormal proteins.

## Introduction

Accumulation and precipitation of damaged or unfolded proteins are associated with many age-related diseases such as Parkinson disease, Huntington’s disease, Alzheimer disease, cataract, and age-related macular degeneration. Therefore, timely removal or repair of the damaged proteins is essential for cellular functions [1-3]. To avoid the accumulation of these damaged proteins, organisms evolved elaborate protein quality control mechanisms that recognize proteins with abnormal structures and either refold them to the normal conformation or target them for degradation [4-7]. The removal of damaged proteins is achieved by proteolytic systems whereas the repair of unfolded proteins is mediated by molecular chaperones.

The ubiquitin-proteasome pathway (UPP) is an important protein quality control mechanism, which selectively recognizes and degrades proteins with abnormal structure [5,8-13]. The UPP is a multi-enzyme cytosolic protein degradation system and is found in all eukaryotic cells. In addition to degrading damaged proteins, the UPP is also involved in conditional degradation of many regulatory intracellular proteins, which control a variety of essential cellular processes including lens differentiation and development [14-20]. In the simplest form, the UPP involves substrate recognition by covalent attachment of ubiquitin to target proteins in a process called ubiquitination and subsequent degradation of the ubiquitin-protein conjugates by the 26S proteasome. In the process of ubiquitination, ubiquitin is first activated by an ubiquitin-activating enzyme (E1) via the formation of a thiol ester bond with E1. The activated ubiquitin is then passed to an ubiquitin conjugating enzyme (Ubc or E2) to which ubiquitin is also linked via a thiol ester bond. The activated ubiquitin is then conjugated to substrates via an ubiquitin ligase (E3). Many E2s and E3s have been identified. The diversity of E2s and E3s is responsible for the substrate specificity and in part for regulation of the ubiquitination process. In addition to degrading regulatory and abnormal cytoplasmic proteins, the UPP also plays a role in the degradation of misfolded endoplasmic reticulum (ER) proteins. In the process of ER-associated degradation (ERAD), misfolded proteins in the ER lumen are specifically recognized and retro-translocated into the cytosol where they are degraded by the UPP [21-23]

Molecular chaperones are another arm of the cellular protein quality control mechanism. Molecular chaperones are a large family of stress-induced proteins that mediate the folding or refolding of other polypeptides but are not components of those final structures [24]. Molecular chaperones are involved in many cellular metabolic processes such as protein folding/refolding, assembly, and degradation [25-29]. The majority of molecular chaperones in the cells are heat-shock proteins that play a key role in preventing the intracellular accumulation and aggregation of misfolded or unfolded proteins, therefore, representing an important defense mechanism against various types of stress [7,30]. Based on cellular locations, molecular chaperones are classified into cytoplasmic chaperones and ER chaperones. In the cytosol, various cellular stresses lead to increased expression of heat-shock proteins [31]. Most of the heat-shock proteins function as molecular chaperones that facilitate the refolding or degradation of damaged or misfolded proteins. Therefore, elevated expression of molecular chaperones in response to stresses may be essential for the cells to prevent the accumulation and aggregation of misfolded or damaged proteins [32]. Accumulation of misfolded or abnormally modified proteins in the ER stimulates the expression of many ER resident proteins, most of which also function as molecular chaperones [33,34]. The increased expression of ER chaperones is often called unfolded protein response (UPR) [35]. UPR may act to increase the protein folding capability of the ER, decrease the rates of protein synthesis, and therefore reduce the burden of protein folding in the ER.

Recent studies indicate that there are functional relationships between the UPP and molecular chaperones. For example, inhibition of the UPP leads to the activation of members of the heat shock factor family [36] and increases the expression of many heat-shock proteins [37,38] and ER chaperones [30]. Some of the heat-shock proteins such as Hsp90 and Hsp70 are required for the degradation of damaged or denatured proteins by the UPP [25,39-42]. We recently demonstrated that the UPP and molecular chaperones recognize the same features of denatured proteins so that the UPP and molecular chaperones work in a competitive manner in eliminating denatured proteins [5]. The balance between the UPP and chaperones appears to be modulated by levels of carboxyl terminus of Hsc-70 interacting protein (CHIP), a co-chaperone with ubiquitin ligase activity [5]. In healthy cells, the delicate balance between the UPP and the chaperones controls damaged proteins to a tolerable level. If one system fails, the other system would be the backup [38,43].

In the UPP, the conserved ubiquitin acts as a degradation signal upon covalent conjugation of multiple ubiquitins to protein substrates [44-46]. Ubiquitin contains seven lysine (K) residues. Some of the seven K residues are essential for the formation of polyubiquitin chains and play a critical role in determining the inter-ubiquitin linkage within the ubiquitin chain, whereas others play roles in maintaining the structure of the ubiquitin conjugates [46]. We have demonstrated that several K6-modified or mutated ubiquitins (K6-biotinylated ubiquitin, K6A, and K6W mutant ubiquitin) behave as dominant negatives, which can inhibit the ubiquitin-mediated degradation [46]. Therefore, the K6W-ubiquitin is a specific inhibitor of the UPP and a valuable reagent for studying the functions of the UPP. We constructed an adenoviral vector for the overexpression of K6W-ubiquitin in the cells and found that the expression of K6W-ubiquitin in lens epithelial cells could inhibit cell cycle progression by impairing UPP-dependent degradation and accumulation of damaged proteins [13,19].

To further elucidate the interactions between UPP and molecular chaperones in the process of protein quality control, we investigated the effect of the expression of dominant negative K6W-ubiquitin on the expression of various cytoplasmic and ER chaperones. We found that the inhibition of UPP by the expression of the K6W-ubiquitin results in upregulation of a broad spectrum of molecular chaperones and stress-regulated proteins. These findings support our hypothesis there is an interaction between molecular chaperones and the UPP in the process of protein quality control. The upregulation of molecular chaperones in response to expression of dominant negative ubiquitin may compensate for the impairment of the UPP in degradation of abnormal proteins.

## Methods

### Cell culture

Human lens epithelial cells (HLEC; SRA 01/04) were obtained from Dr. Venkat Reddy, Oakland University, Rochester, MI [47] and cultured at 37 °C in the presence of 5% CO_2_ in Dulbecco’s modified Eagle medium (DMEM; Gibco/ BRL, Gaithersburg, MD) supplemented with 10% fetal bovine serum (HyClone, Logan, UT) and 100 U/ml penicillin G and 100 mg/ml streptomycin.

### Expression of K6W-ubiquitin in human lens epithelial cells

HLEC were grown to 100% confluence and then infected with replication deficient adenoviruses that encode green fluorescent protein (GFP) only (the control) or with GFP and K6W-ubiquitin. The adenoviruses were generated using the AdEasy^TM^ adenoviral vector system (Stratagene, La Jolla, CA) as described previously [46]. Adenoviruses were purified by cesium density gradient centrifugation. The titer of the adenovirus stock was 2x10^12^ plaque forming units (pfu)/ml, and the cells were infected by adenovirus at an average concentration of 1,000 pfu/cell. As judged by GFP expression, greater than 90% of the cells were infected and expressed recombinant proteins. The cells were harvested 48 h after infection with adenoviruses for extracting total RNA and with total proteins for evaluating the expression of molecular chaperones.

### Inhibition of proteasome, endoplasmic reticulum stress, and heat shock stress of human lens epithelial cells

HLEC were grown to confluence and then cultured in a fresh medium with or without MG132, a proteasome inhibitor and tunicamycin, an inhibitor of N-linked glycosylation. MG132 was prepared in DMSO as 10 mM stock solution and diluted to 10 μM in the culture medium immediately before use. The stock solution of tunicamycin (5 mg/ml) was prepared in DMSO and added to medium at a final concentration of 5 μg/ml. The same amount of DMSO was added into the medium of the control group. After 6 h of incubation with MG132 or tunicamycin, the cells were harvested for extraction of total RNA or total proteins. For treatment of cell culture with heat shock, confluent HLEC were incubated at 45 °C for 0.5 h or 1 h and then incubated at 37 °C for 1 h. The cells were then harvested for total RNA and protein extraction.

### Real time polymerase chain reaction analysis

Total RNA was extracted from HLEC using the RNeasy mini kit (Qiagen, Valencia, CA). Total RNA (2 μg) from each sample were used for reverse transcription using SuperScript First-Strand Synthesis System for reverse transcription polymerase chain reaction (RT–PCR; Invitrogen, Carlsbad, CA). The sequences of the primers used for real-time RT–PCR are summarized in Table 1. Real-time RT–PCR analysis was conducted on Stratagene Mx4000 multiplex quantitative PCR system using SYBR Green PCR master mix (Qiagen) according to the manufacturer’s instructions. The expression levels of the genes of interest were normalized using *GAPDH* as a housekeeping gene.

**Table 1:** Primers for real-time reverse transcription polymerase chain reaction.

**Gene**	**Forward primer**	**Reverse primer**
αA-crys	5′-CGGGACAAGTTCGTCATCTT-3′	5′-GCAGACAGGGAGCAAGAGAG-3′
αB-crys	5′-TTCTTCGGAGAGCACCTGTT-3′	5′-TTTTCCATGCACCTCAATCA-3′
Hsp27	5′-ACGAGCATGGCTACATCTCC-3′	5′-CTTTACTTGGCGGCAGTCTC-3′
Hsp40	5′-CTCAGTTTCATGCTGCCGTA-3′	5′-TTGCTGCAGTGAAGTCCATC-3′
Hsp70	5′-CGACCTGAACAAGAGCATCA-3′	5′-AAGATCTGCGTCTGCTTGGT-3′
Hsc70	5′-GGAGGTGGCACTTTTGATGT-3′	5′-AGCAGTACGGAGGCGTCTTA-3′
Hsp90A	5′-GCCTCTGGTGATGAGATGGTTTC-3′	5′-TGTTTCCGAAGACGTTCCACAA-3′
Hsp90B	5′-GAGCTGCTGCGCTATCATACCT-3′	5′-AAAAGCTGAGTTGGCCACCT-3′
Grp75	5′-TCTGGACTGAATGTGCTTCG-3′	5′-ATCCCCATTTGTGGATTTCA-3′
Grp78	5′-TAGCGTATGGTGCTGCTGTC-3′	5′-TTTGTCAGGGGTCTTTCACC-3′
Grp94	5′-TGGGAAGAGGTTCCAGAATG-3′	5′-GTTGCCAGACCATCCGTACT-3′
PDI	5′-AAGCTCAGCAAAGACCCAAA-3′	5′-CACTTAATTCACGGCCACCT-3′
CHOP	5′-TGCCTTTCTCTTCGGACACT-3′	5′-TGTGACCTCTGCTGGTTCTG-3′
Hsp60	5′-CATTCCAGCCTTGGACTCAT-3′	5′-TCACAACCTTTGTTGGGTCA-3′
GAPDH	5′-ATCACCATCTTCCAGGAGCGA-3′	5′-CCTTCTCCATGGTGGTGAAGAC-3′

The expression levels of the genes of interest were normalized using *GAPDH* as a housekeeping gene.

### Western blotting

The cells were lysed with SDS-gel loading buffer. After boiling at 100 °C for 3 min, 10 µg (for determining Hsp70, Hsp90, and β-actin) or 50 µg (for determining other chaperones) of total cellular proteins were resolved by SDS–PAGE using 12% gels. Levels of Hsp70, Hsp90, Grp75, and Grp78 were determined by western blotting. β-actin was used as a protein loading control. Antibodies to Hsp70 and β-actin were purchased from Sigma (St. Louis, MO), antibodies to Hsp90 were purchased from Cell Signaling (Danvers, MA), and antibodies to Grp75 and Grp78 were purchased from Santa Cruz (Santa Cruz, CA).

## Results

### Expression of K6W-ubiquitin in human lens epithelial cells increases the mRNA levels of a broad spectrum of cytoplasmic and endoplasmic reticulum chaperones in human lens epithelial cells

**Figure 1:**
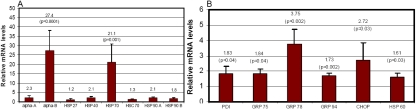
Expression of K6W-ubiquitin in lens epithelial cells upregulates cytoplasmic, endoplasmic reticulum, and mitochondrial chaperones. Lens epithelial cells were infected with the adenovirus encoding K6W-ubiquitin or the control adenovirus for 48 h. Total RNA was extracted, and the levels of mRNA for these chaperones were determined by real-time RT–PCR using *GAPDH* as a reference. The level of mRNA for these chaperones in cells infected by the control adenovirus was arbitrarily designated as 1. The level of mRNA for these chaperones in cells that express K6W-ubiquitin were normalized with the control and expressed as relative levels. Panel **A** shows the expression of cytoplasmic chaperones. Panel **B** shows the expression of ER chaperones, an ER stress-related factor, and a mitochondrial chaperone.

We previously demonstrated that K6W-ubiquitin is a dominant negative inhibitor of the UPP and that the expression of K6W-ubiquitin in HLEC stabilizes typical substrates for the UPP and delays the cell cycle progress [19,46]. In this study, we investigated the effect of K6W-ubiquitin on the expression of cytoplasmic and ER chaperones. Among the cytoplasmic chaperones, mRNA levels for αB-crystallin and Hsp70 increased 27 fold and 21 fold, respectively, after the expression of K6W-ubiquitin (Figure 1A). The mRNA levels for αA-crystallin, Hsp40, and Hsp90 increased about twofold in response to the expression of K6W-ubiquitin whereas mRNA levels for Hsp27 and Hsc70 did not change significantly (Figure 1A). mRNA levels for ER chaperones, including protein disulfide isomerase (PDI), and for glucose regulated proteins (Grp75, Grp78, and Grp94) increased from 1.7 to 3.7 fold in response to the expression of K6W-ubiquitin (Figure 1B). The ER stress-induced transcription factor, CHOP, and the mitochondrial chaperone, Hsp60, also increased 2.7 and 1.6 fold, respectively (Figure 1B). These data indicate that the expression of K6W-ubiquitin in HLEC not only increased the expression of cytoplasmic chaperones but also increased the ER and mitochondrial chaperones.

**Figure 2:**
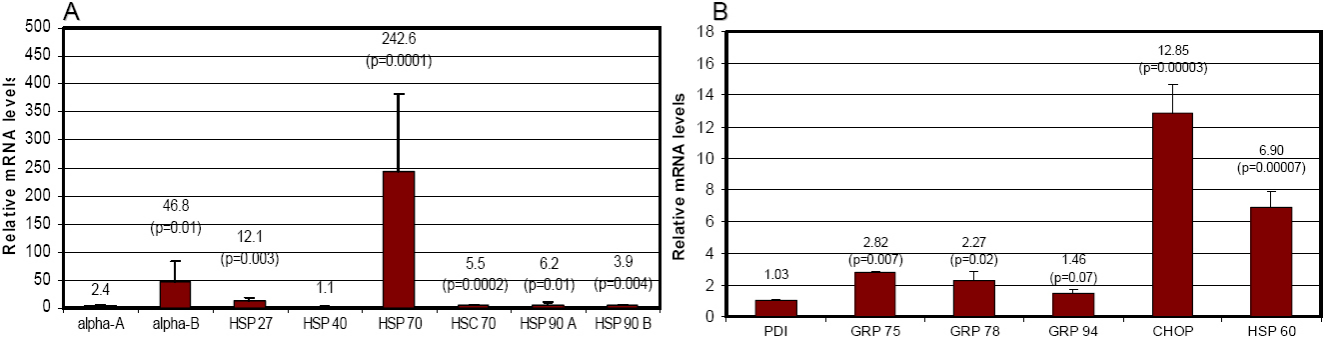
Proteasome inhibition upregulates cytoplasmic, endoplasmic reticulum, and mitochondrial chaperones. Lens epithelial cells were treated with or without 10 µM MG132 for 6 h. Total RNA was extracted, and the level of mRNA for these chaperones was determined by real-time RT–PCR using *GAPDH* a reference. The level of mRNA for these chaperones in the cells not treated with MG132 (the control) was arbitrarily designated as 1. The levels of mRNAs for these chaperones in MG132-treated cells were normalized with the control and expressed as relative levels. Panel **A** shows the expression of cytoplasmic chaperones. Panel **B** shows the expression of ER chaperones, an ER stress-related factor, and a mitochondrial chaperone.

To confirm that the upregulation of molecular chaperones in response to the expression of K6W-ubiquitin is due to the impairment of the UPP, we determined the effect of the proteasome inhibitor on the expression of these chaperones. We found that proteasome inhibition also increased the expression of these cytoplasmic and ER chaperones, and the extents of increase are more dramatic than that caused by the expression of K6W-ubiquitin. The mRNA levels for αB-crystallin and Hsp70 increased 42 and 242 fold, respectively, after incubation with MG132 for 6 h (Figure 2A). The mRNA levels for αA-crystallin, Hsp27, Hsc70, and Hsp90 increased from 2.4 to 12-fold in response to proteasome inhibition, but the mRNA level for Hsp40 did not change (Figure 2A). Among the ER chaperones, mRNA levels for Grp75, Grp78, and Grp94 increased from 1.46 to 2.8 fold, but the mRNA level for PDI did not change in response to proteasome inhibition (Figure 2B). The ER stress-related transcription factor, CHOP, and the mitochondrial chaperone, Hsp60, also increased 12.8 and 6.9 fold, respectively, after incubation with MG132 (Figure 2B). These data confirmed that impairment of the UPP leads to upregulation of a broad spectrum of cytoplasmic, ER, and mitochondrial chaperones.

### Heat shock increases the expression of Hsp70 and some endoplasmic reticulum chaperones in human lens epithelial cells

**Figure 3:**
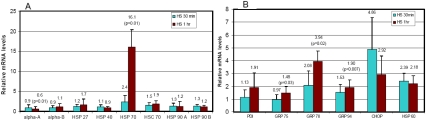
Heat shock increases the expression of Hsp70 and some endoplasmic reticulum and mitochondrial chaperones. Lens epithelial cells were cultured at 45 °C for 0, 30, or 60 min and then cultured at 37 °C for 1 h. Total RNA was extracted, and levels of mRNA for these chaperones were determined by real-time RT–PCR using *GAPDH* as a reference. The level of mRNA for these chaperones in cells cultured in 37 °C (control) was arbitrarily designated as 1. The levels of mRNAs for these chaperones in heat-shocked cells were normalized with the control and expressed as relative levels. Panel **A** shows the expression of cytoplasmic chaperones. Panel **B** shows the expression of ER chaperones, an ER stress-related factor, and a mitochondrial chaperone.

To begin elucidating the molecular mechanisms of the upregulation of molecular chaperones induced by the impairment of the UPP, we compared the expression patterns of these cytoplasmic and ER chaperones upon impairment of the UPP and in response to heat shock. We found that HLEC are relatively resistant to heat shock as incubation of the cells at 43 °C for 1 h had little effect on the expression of any of these chaperones (data not shown). Incubation of the cells at 45 °C for 30 min only marginally increased the expression of Hsp70 and some ER chaperones (Figure 3). In contrast, incubation of HLEC cells at 45 °C for 1 h increased the expression of Hsp70 16-fold and the mRNA levels for Hsp27 and Hsp70 increased slightly (less than twofold). The other cytoplasmic chaperones either did not change or decreased (αA-crystallin) under these conditions (Figure 3A). The incubation of HLEC at 45 °C also increased the expression of some ER chaperones, an ER stress-related transcription factor, and mitochondrial chaperones (Figure 3B). These data show that heat shock selectively triggers the expression of some cytoplasmic and ER chaperones but not as broad as the impairment of the UPP, indicating that impairment of the UPP and heat shock use a similar but not identical mechanism to activate the expression of cytoplasmic and ER chaperones.

### Endoplasmic reticulum stress results in the upregulation of endoplasmic reticulum chaperones and the downregulation of cytoplasmic chaperones

**Figure 4:**
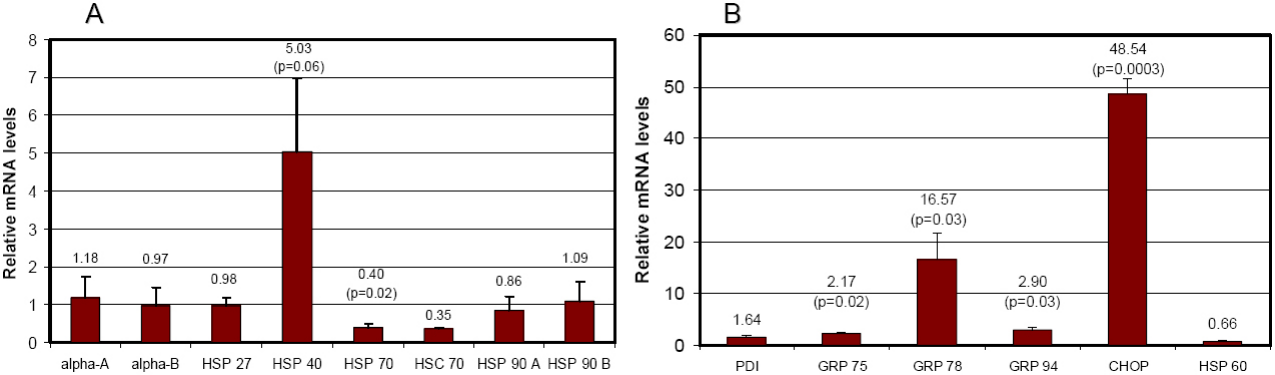
Disruption of endoplasmic reticulum function in lens epithelial cells upregulates Hsp40 and many endoplasmic reticulum chaperones. Lens epithelial cells were treated with or without 5 µg/ml tunicamycin for 6 h. Total RNA was extracted, and levels of mRNA for these chaperones were determined by real-time RT–PCR using *GAPDH* as a reference. The level of mRNA for these chaperones in cells not treated with tunicamycin (control) was arbitrarily designated as 1, and the level of mRNA for these chaperones in tunicamycin-treated cells was normalized with the control and expressed as relative levels. Panel **A** shows the expression of cytoplasmic chaperones. Panel **B** shows the expression of ER chaperones, an ER stress-related factor, and a mitochondrial chaperone.

The data presented above indicate that heat shock not only increases the expression of cytoplasmic chaperones but also enhances the expression of some ER chaperones, indicating that heat shock may cause both cytoplasmic stress and ER stress. To further determine the regulation of cytoplasmic and ER chaperones, we imposed ER stress via inhibiting N-linked glycosylation by tunicamycin. We found that treatment of HLEC with tunicamycin resulted in the downregulation of cytoplasmic chaperones, Hsp70 and Hsc70, but increased the expression of Hsp40 (Figure 4A). The expression of mitochondrial chaperone, Hsp60, was also downregulated by the treatment with tunicamycin. In contrast, treatment with tunicamycin resulted in upregulation of all ER chaperones (Figure 4B). The mRNA levels for CHOP, an ER stress-related factor, increased 48 fold in response to tunicamycin treatment (Figure 4B). These data indicate that the expressions of cytoplasmic chaperones and ER chaperones are controlled by distinct transcription factors in HLEC.

### Expression of K6W-ubiquitin, proteasome inhibition, and heat shock increase Hsp70 protein in human lens epithelial cells

**Figure 5:**
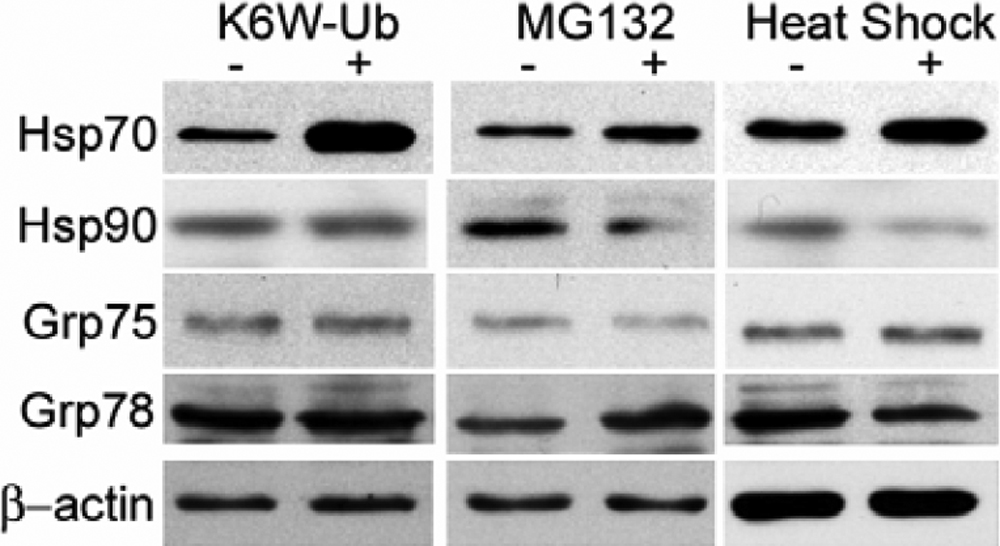
Expression of K6W-ubiquitin, proteasome inhibition, and heat shock increase the Hsp70 protein in human lens epithelial cells. Lens epithelial cells were infected with the adenovirus encoding K6W-ubiquitin or the control adenovirus for 48 h, treated with or without MG132 for 6 h, or incubated at 45 °C for 1 h and recovered at 37 °C for 1 h. Total proteins were collected in a SDS-loading buffer. Levels of Hsp70, Hsp90, Grp75, and Grp78 were determined by western blotting using respective antibodies. The levels of β-actin were also determined by western blotting and used as a protein loading control.

To further investigate if the increased mRNAs of these molecular chaperones are translated into proteins, we determined the effects of K6W-ubiquitin expression and proteasome inhibition on the protein levels of αB-crystallin, Hsp70, Hsp90, Grp75, and Grp78 by western blotting. We found that levels of αB-crystallin HLEC are below the detecting limit. Although the mRNA levels for αB-crystallin increased more than 20-fold upon the expression of K6W-ubiquitin, the protein of αB-crystallin was not detectable in the cells (data not shown). The proteins of Hsp70, Hsp90, Grp75, and Grp78 are readily detected in HLEC even under non-stress conditions. The expression of K6W-ubiquitin significantly increased the protein levels of Hsp70 (Figure 5). However, the protein levels of Hsp90, Grp75, and Grp78 were not affected by the expression of K6W-ubiquitin (Figure 5), although their mRNA levels increased under these conditions (Figure 1). Consistent with the expression of K6W-ubiquitin, the inhibition of the proteasome and heat shock treatment also increased the protein levels of Hsp70 in HLEC (Figure 5). In contrast, protein levels of Hsp90, Grp75, and Grp78 did not change significantly under any of these conditions (Figure 5). These data indicate that increased levels of mRNA in response to K6W-ubiquitin expression or other stresses were only partially translated into proteins at the time we collected the cells. It may take longer for the cells to translate these mRNA into proteins.

## Discussion

The UPP and molecular chaperones are the two major components of the cellular protein quality control mechanism. Whereas the UPP degrades the damaged proteins, the chaperones either prevent the aggregation of damaged proteins or refold denatured proteins. We demonstrated previously that the UPP and molecular chaperones compete for denatured proteins [5]. While the enhancement of ubiquitination reduces the chaperone-mediated renaturation, increasing chaperone activity reduced the UPP-mediated degradation of denatured proteins [5]. To further study the functional relationship between the UPP and molecular chaperones, we studied the effects of UPP impairment on the expression of cytoplasmic, ER, and mitochondrial chaperones. We found that the expression of K6W-ubiquitin in HLEC leads to the upregulation of a broad spectrum of both cytoplasmic and ER chaperones. Among the cytoplasmic chaperones, mRNA for αB-crystallin, Hsp70, and Hsp90 increased 27 fold, 21 fold, and twofold, respectively, in response to the expression of K6W-ubiquitin (Figure 1A). Among the ER stress proteins, mRNA levels of Grp78 and CHOP increased 3.7 fold and 2.7 fold, respectively (Figure 1B). Other cytoplasmic and ER chaperones are also upregulated in response to the expression of K6W-ubiquitin but to a less extent.

The predominant upregulation of Hsp70 and αB-crystallin in response to K6W-ubiquitin suggests that Hsp70 and αB-crystallin play an important role in the quality control of cytoplasmic proteins. It is known that Hsp70 is one of the major chaperones that can refold denatured proteins in an ATP-dependent manner. Although αB-crystallin is not able to refold denatured proteins, it can hold and deliver the denatured proteins to Hsp70 and other chaperones for refolding [48,49].

Consistent with upregulation of cytoplasmic and ER chaperones upon the expression of K6W-ubiquitin, inhibition of the proteasome by MG132 also induced the expression of both cytoplasmic and ER chaperones (Figures 2A,B). These data support our hypothesis that the UPP and chaperones are interrelated. The upregulation of these chaperones may compensate for the impairment of the UPP and prevent the accumulation and aggregations of damaged proteins in the cells via increasing the cellular capacity of refolding of denatured proteins.

It has been reported that inhibition of the UPP increases the expression of many cytoplasmic chaperones in many cell types [30,38,50-52]. However, the information regarding the effect of impairment of the UPP on the expression of ER chaperones is scarce and controversial. An early study showed that inhibition of the UPP in Madin-Darby canine kidney cells induced the expression of both cytoplasmic and ER chaperones [30] yet a recent study indicated that inhibition of the proteasome in HepG2 cells only induced the expression of cytoplasmic chaperones but not ER stress proteins [52]. This study shows that in lens epithelial cells, both cytoplasmic and ER chaperones are upregulated by inhibition of the UPP. It seems that the UPP-dependent regulation of cytoplasmic chaperones is universal, but the regulation of ER chaperones is cell-type specific. To begin investigating the mechanisms by which the UPP regulates the expression of cytoplasmic and ER chaperones, we compared the expression patterns of these chaperones induced by inhibition of the UPP by heat-shock stress and by the interruption of ER functions. We found that heat-shock stress induced the expression of Hsp70 but not other cytoplasmic chaperones in the lens cells (Figure 3A). This is different from other cell types and tissues [53]. One possible explanation for the difference is that this transformed cell line is relatively resistant to heat-shock stress. It may need a higher temperature or longer time to induce the expression of these chaperones in this cell line. However, we found that heat-shock stress induced the expression of many ER chaperones (Figure 3B). Disruption of ER functions such as the inhibition of glycosylation by tunicamycin induced the expression of many ER chaperones (Figure 4B) but not classic cytoplasmic chaperones (Figure 4A). In fact, the expression of Hsp70 and Hsc70 decreased upon treatment with tunicamycin (Figure 4A). Hsp40 is the only cytoplasmic chaperone expressed that was induced by treatment with tunicamycin. These data suggest that the expression of cytoplasmic chaperones and ER chaperones are regulated by different signals. Impairment of the UPP and heat-shock stress activated both signals that induce the expression of cytoplasmic and ER chaperones, but the inhibition of glycosylation by tunicamycin only triggered the signal that induces the expression of ER chaperones.

How does impairment of the UPP trigger the expression of these chaperones? It is generally believed that accumulation of misfolded or abnormal proteins is the primary signal for the induction of chaperones [54]. Whereas cytosolic accumulation of abnormal proteins triggers the expression of cytoplasmic chaperones, the accumulation of abnormal proteins in the ER lumen leads to ER stress and the expression of ER chaperones. Many abnormal proteins in the cytoplasm are degraded by the UPP [4,12,13,55-57]. Impairment of the UPP will likely increase the levels of abnormal proteins and subsequently trigger the expression of cytoplasmic chaperones. Another possible mechanism for the induction of cytoplasmic chaperones in response to impairment of the UPP is the stabilization of heat-shock factors (HSFs). HSFs are transcription factors that control the transcription of many cytoplasmic chaperones. Mammalian cells express a family of HSFs, and their activities are regulated by diverse stress conditions to coordinate the inducible expression of heat shock genes including molecular chaperones [58]. HSF1 is expressed ubiquitously and activated by various stresses that elevate levels of abnormal proteins. The expression and activity of HSF2 have been associated with development and differentiation. Although HSF2 is not activated by heat shock, it is activated by proteasome inhibition. The levels and DNA-binding activity of HSF2 are induced upon exposure of cells to various proteasome inhibitors such as hemin, MG132, and lactacystin [37]. It is suggested that HSF2 is a substrate for the UPP and that impairment of the UPP results in accumulation and activation of HSF2 [37]. The downstream effect of HSF2 activation by proteasome inhibitors is the induction of the same set of heat-shock proteins that are triggered by HSF1 upon heat-shock stress [37]. Consistent with the role of HSF2 in the induction of cytoplasmic chaperones in response to impairment of the UPP, it has been reported that inhibition of proteasome increased the levels of HSF2 and HSF4 in lens epithelial cells [51]. It is important to further investigate the mechanism by which the UPP regulates the expression of molecular chaperones. Another potential mechanism is that the activation of HSF requires ubiquitination. In another word, ubiquitinated HSF may be the active form. We propose to test this possibility in future studies by determining the effect of altering the ubiquitination process such as knockdown or inhibition of an ubiquitin-activating enzyme on the expression of molecular chaperones.

The quality of secretory and transmembrane proteins is controlled in the endoplasmic reticulum (ER), and only correctly folded proteins are allowed to reach their final destination. Protein quality control in the ER is achieved by two different mechanisms, which direct proteins to opposing outcomes [59]. The first is the productive folding mechanism, which is assisted by several ER chaperones. The second is the ERAD mechanism by which unfolded or misfolded proteins in the ER are recognized and retro-translocated to the cytosol for degradation by the UPP. Under various stress conditions, elevated levels of unfolded proteins in the ER trigger the expression of ER chaperones and ERAD components in a process called unfolded protein response (UPR) to enhance the capacity of productive folding and degradation mechanisms [60]. There are three major ER resident transmembrane proteins, PERK, ATF6, and IRE1, as proximal signal sensor in mammalian cells [61]. Molecular chaperones also play regulatory roles in the UPR signaling pathway. The best characterized is the ER chaperone, Grp78, which directly interacts with all three ER stress sensors, PERK, ATF6, and IRE1, and maintains them in inactive forms in unstressed cells [35]. When levels of misfolded proteins increase, they compete with ER stress sensors for Grp78. The free ER stress sensors allow the activation and transduction of the unfolded protein signals across the ER membrane to the nucleus and induce the expression of ER chaperones and ERAD components. The expression of K6W-ubiquitin or the inhibition of the proteasome may decrease the efficiency of the ERAD system and result in the accumulation of abnormal proteins in the ER lumen and subsequently activating the UPR and increasing the expression of ER chaperones. The data presented here demonstrate that the function of the UPP in HLEC is closely related to ER stress, suggesting that HLEC have an active ERAD system. Consistent with the hypothesis, lens cells have an active UPR system and many cataractogenic agents also activate UPR in lens epithelial cells [62-64]. However, the role of ERAD in protein homeostasis in lens epithelial cells remains to be determined.

The extents to which the mRNA of these chaperones increased in response to different stressors were much greater than that of the increase in protein levels. We only detect a consistent increase in Hsp70 at protein levels (Figure 5). It is known that the abundance of mRNA and the levels of proteins are not always correlated [65,66]. Whereas the levels of mRNA are mainly controlled by the transcription activity and mRNA stability, the levels of proteins reflect mRNA level, translation efficiency, posttranslational modification, and degradation. Alteration in any of these steps may affect the overall protein quality control capability.

Consistent with our working model that the UPP and chaperones work in a parallel or competitive manner in eliminating abnormal proteins, the induction of cytoplasmic and ER chaperones in response to the impairment of the UPP may be a compensating mechanism to prevent the accumulation of damaged proteins in the cells under stress conditions. The induction of these chaperones may also provide a protective effect against other types of stresses. For example, pretreatment of the kidney cells with proteasome inhibitors results in an induction of cytoplasmic and ER chaperones as well as thermal tolerance [30]. In lens epithelial cells, proteasome inhibition not only increased the expression of many cytoplasmic chaperones, but it also protects the cells from apoptosis induced by interferon-gamma [51]. Pretreatment of lens epithelial cells with quercetin, which diminishes the induction of chaperones, abolished the protective effect of the proteasome inhibitor against apoptotic cell death induced by interferon-gamma [51]. The interrelationship between the UPP and the chaperones appears to be an important mechanism adapted by the cells to cope with various types of stress. When the function of one system is compromised, the other system would be upregulated and would compensate for the loss of function. In healthy cells, the delicate balance between the UPP and the chaperones controls damaged proteins to a tolerable level. However, upon aging or severe stress, functions of both the UPP and the chaperones may be overwhelmed or compromised. For example, we found that proteasome in lens epithelial cells and other types of cells were inactivated by prolonged oxidative stress (unpublished data). Severe oxidation or other stressors may also impair the chaperone activity of heat shock proteins [67]. Dysfunction of the protein quality control mechanism may be causally related to the accumulation of damaged and aggregated proteins in the cells. Therefore, means of protecting the function of the protein quality control system may be used to prevent several age-related diseases such as cataract.
